# Development and evaluation of a nomogram model for predicting prolonged hospitalization after spinal tuberculosis focus decompression, fusion, and internal fixation surgery

**DOI:** 10.1186/s40001-025-03805-8

**Published:** 2026-01-13

**Authors:** Xu-Feng Jia, Qing-Zhong Zhou, Miao Long, Yun-Long Zhou, Shao-Hua Wang, Da-Xiong Feng

**Affiliations:** 1 Department of Orthopaedics, The Affiliated Hospital of Southwest Medical University, No.25 Taiping Street, Jiangyang District, Luzhou, 646000 Sichuan China; 2 Department of Orthopaedics, Jianyang Hospital, Chengdu Medical College People’s Hospital of Jianyang City, Jianyang, Chengdu, 641400 Sichuan China; 3 Department of Ultrasound, Jianyang Hospital, Chengdu Medical College People’s Hospital of Jianyang City, Jianyang, Chengdu, 641400 Sichuan China; 4 Department of Orthopedics 1, Leshan People’s Hospital, Leshan, 614099 Sichuan China; 5 Department of Trauma, Jianyang Hospital, Chengdu Medical College People’s Hospital of Jianyang City, Jianyang, Chengdu, 641400 Sichuan China

**Keywords:** Spinal tuberculosis, Prolonged hospitalization, Nomogram, Decompression, Risk prediction

## Abstract

**Background:**

Spinal tuberculosis remains a significant clinical challenge in high-prevalence regions. Despite advances in medical treatment, surgical interventions, such as debridement, decompression, and fusion, are often required, but their complexity increases perioperative morbidity and prolongs hospitalization. Identifying preoperative predictors of extended hospital stay may improve patient management and resource allocation.

**Methods:**

In this retrospective study, 256 patients with confirmed spinal tuberculosis who were treated between January 2021 and December 2024 were included. Prolonged hospitalization was defined as a postoperative stay > 21 days. Univariate and multivariate logistic regression analyses were performed, and significant predictors were integrated into a nomogram. Model performance was evaluated by receiver operating characteristic (ROC) analysis, calibration plots with bootstrap resampling, and decision curve analysis (DCA).

**Results:**

The patients were divided into a modeling group (*n* = 170) and a validation group (*n* = 86). Baseline characteristics were comparable between groups. Multivariate analysis identified increasing age (OR 1.042, 95% CI 1.013–1.071, *P* = 0.005), concomitant TB at other sites (OR 2.875, 95% CI 1.168–7.100, *P* = 0.022), and a higher preoperative American Society of Anesthesiologists score (OR 1.537, 95% CI 1.010–2.340, *P* = 0.046) as independent predictors of prolonged hospitalization. The nomogram demonstrated good discriminative ability (AUC: 0.771 and 0.718) and satisfactory calibration (corrected C index 0.787; Hosmer–Lemeshow *P* = 0.895). DCA confirmed its clinical utility.

**Conclusions:**

Advanced age, extra-spinal TB involvement, and elevated preoperative ASA score are significant predictors of prolonged hospitalization after spinal TB surgery. The developed nomogram is a practical tool for preoperative risk assessment, warranting further prospective multicenter validation.

**Supplementary Information:**

The online version contains supplementary material available at 10.1186/s40001-025-03805-8.

## Introduction

Spinal tuberculosis (TB), also known as Pott’s disease, is the most common manifestation of skeletal tuberculosis and a significant extra-pulmonary form of *Mycobacterium tuberculosis* infection [1]. It poses considerable clinical challenges in regions with high TB prevalence, frequently causing spinal deformities, neurological deficits, and prolonged hospitalization if diagnosis and treatment are delayed [2–4]. Despite advancements in pharmacological therapy, surgical intervention, typically involving debridement, decompression, fusion, and internal fixation, is often necessary when conservative management is ineffective or when neurological impairment and spinal instability occur. These surgical procedures aim to eradicate the infectious focus, alleviate neural compression, and re-establish spinal stability. Nevertheless, due to their complexity, such interventions carry substantial perioperative morbidity and frequently lead to prolonged hospitalization, which increases healthcare costs and predisposes patients to nosocomial complications. Thus, accurate predictive tools to identify patients at elevated risk for extended postoperative hospitalization are urgently needed [5, 6].

Recent years have seen a growing emphasis on the use of predictive models to improve clinical decision-making and optimize resource allocation for surgical patients. Among these models, nomograms have become valuable tools that integrate multiple prognostic variables to estimate individual risk. Their graphical format allows for easy bedside application, enabling clinicians to stratify patients based on risk and tailor perioperative management accordingly. The multifactorial nature of prolonged hospitalization after spinal TB surgery reflects the interaction of patient-related factors, such as comorbidities and nutritional status, with procedure-related factors, including the extent of surgical debridement and the degree of spinal instability [1, 7]. In addition, factors like the duration of surgery, intraoperative blood loss, and postoperative complications contribute to the variability in hospital stay length. Thus, developing a robust predictive model requires a systematic assessment of these variables to identify the most significant predictors of prolonged hospitalization. An accurate nomogram that quantifies these risks can be a valuable tool in preoperative planning and postoperative care, enabling early identification of high-risk patients and the implementation of targeted interventions to prevent potential complications [8, 9]. However, very few studies have established a validated, preoperative prediction model specifically targeting prolonged hospitalization following spinal tuberculosis decompression and fusion surgery [10, 11]. Length of stay is a critical outcome that reflects disease severity, surgical recovery, and resource utilization, yet it remains underexplored in this population [12, 13]. Furthermore, existing research often relies on postoperative variables, limiting its applicability for preoperative decision-making, or lacks a clinically intuitive tool that can be readily applied at the bedside.

The present study aims to develop and evaluate a nomogram model that predicts the risk of prolonged hospitalization following spinal TB focus decompression, fusion, and internal fixation surgery. By retrospectively analyzing patients who underwent this procedure, the study seeks to identify independent risk factors that influence the duration of hospitalization. Multivariate logistic regression analysis will be used to quantify the relationship between candidate variables and prolonged hospital stay, with the resulting model presented in the form of a nomogram. This approach is designed to facilitate individualized risk assessment, thereby improving clinical outcomes and optimizing the use of healthcare resources.

## Methods

### Study design

This retrospective study encompassed patients with confirmed spinal tuberculosis managed at our institution from January 2021 to December 2024. The study’s design and reporting adhered to the STROBE (Strengthening the Reporting of Observational Studies in Epidemiology) guidelines [14]. Inclusion criteria required a definitive diagnosis based on clinical evidence (symptoms such as back pain, fever, or neurological deficits consistent with spinal tuberculosis), imaging findings (radiological evidence of lesions or destruction in the spine observed through X-ray, MRI, or CT scans), microbiological evidence (positive results from cultures of tissue or fluids, such as sputum or biopsy specimens, showing *Mycobacterium tuberculosis*), or histopathological evidence (tissue biopsy showing granulomatous inflammation consistent with tuberculosis). Additional criteria included standardized decompression, fusion, and internal fixation surgery, age 18 years or older, and the availability of complete clinical data. Exclusion criteria included a history of previous spinal surgery, incomplete clinical records, severe systemic comorbidities, and an immunocompromised status. A total of 256 patients meeting these stringent criteria were enrolled. Informed consent was obtained from all subjects. This study was approved by the hospital’s ethics committee and conducted in accordance with relevant guidelines and the Declaration of Helsinki. All data were handled confidentially, and personal identifiers were removed prior to analysis to ensure participant privacy. All patients underwent standardized decompression, fusion, and internal fixation surgery, with surgical indications based on clinical guidelines from the British Infection Society for tuberculosis of the central nervous system and the American College of Surgeons National Surgical Quality Improvement Program [15, 16]. Surgical indications included failure of medical therapy, neurological deterioration, significant vertebral destruction, and the presence of spinal instability. Surgeries were performed by a single surgical team.

### Definition of prolonged hospitalization in patients with spinal tuberculosis

Based on evidence from prior studies and our institution's clinical experience, the duration of postoperative hospitalization was quantified using the median value derived from the study population [12, 17, 18]. In this investigation, a postoperative hospital stay exceeding 21 days was designated as prolonged hospitalization, whereas a stay of 21 days or fewer was considered within the normal range. This threshold was determined through statistical analysis of the distribution of hospital stay durations and is consistent with established criteria in similar studies.

### Data collection

Data collection encompassed a comprehensive array of variables extracted from patient clinical records. General clinical data included age, height, weight, sex, body mass index (BMI), and history of alcohol consumption and smoking. Disease-specific parameters comprised the anatomical location of lesions, duration of postoperative hospitalization, presence of spinal cord injury, concurrent extra-pulmonary tuberculosis, evidence of bony destruction, intraoperative blood loss, transfusion requirements, and the occurrence of postoperative complications. Additional intraoperative and postoperative factors, such as drainage tube removal time, surgical duration, preoperative and postoperative American Society of Anesthesiologists (ASA) scores, surgical incision length, and the presence of purulent discharge were also recorded. Moreover, laboratory investigations focused on the detection of postoperative hypoalbuminemia and anemia. In addition, two functional status indicators were assessed. Oswestry disability index (ODI) was used to evaluate the degree of functional impairment related to spinal pathology prior to surgery. Preoperative ambulation ability was also recorded and categorized as either independent or assisted ambulation, reflecting patients’ baseline mobility status.

### Statistical analysis

For model construction and validation, the population was approximately split in a 2:1 ratio into a modeling group (*n* = 170) and a validation group (*n* = 86). Continuous variables following a normal distribution were expressed as mean ± standard deviation, while those not conforming to normality were presented as medians with interquartile ranges (Q1, Q3). Group comparisons for continuous data were performed using either the *t* test or the Wilcoxon rank-sum test as appropriate. Categorical variables were expressed as percentages, with group comparisons conducted using Fisher’s exact test or chi-square test; a two-tailed *P* value of < 0.05 was considered statistically significant. Univariate logistic regression analysis was initially employed to evaluate potential risk factors associated with prolonged hospitalization following debridement, fusion, and internal fixation in spinal tuberculosis patients. Variables identified as significant were subsequently incorporated into a multivariate logistic regression model using a stepwise selection method, with model selection guided by the Akaike information criterion (AIC); lower AIC values indicated a superior model. The discriminative ability of the final model was quantified by calculating the area under the receiver operating characteristic curve (AUC). Model calibration was evaluated using the Hosmer–Lemeshow test, with a non-significant result indicating adequate agreement between predicted and observed probabilities. Statistical analysis was performed using SPSS version 26.0 and R version 4.3.3. The development and validation of the nomogram, as well as the generation of ROC curves, calibration plots, and decision curve analysis (DCA), were facilitated by the rms, pROC, and rmda packages in R. To assess multi-collinearity among predictors, we calculated variance inflation factors (VIFs) and examined pairwise correlations. Variables with VIF > 5 or |*r*|> 0.7 were excluded to prevent collinearity from inflating effect estimates. Sensitivity analyses were conducted to evaluate model robustness by refitting the multivariable logistic regression model with adjustment for operative time and intraoperative blood loss, and by repeating model development after excluding patients in the highest quartile of operative time.

## Results

### Baseline demographic, clinical, and perioperative characteristics of the modeling and validation groups

The study population consisted of 256 patients, with 170 patients in the modeling group and 86 patients in the validation group. Of these, 45 (26.47%) patients in the modeling group and 31 (36.05%) patients in the validation group had prolonged hospitalization (> 21 days). The groups were comparable in terms of baseline demographic and clinical characteristics. The median age in the modeling group was similar to that in the validation group, with no statistically significant difference (*P* = 0.312). The distributions of body mass index, gender, smoking history, and alcohol consumption were comparable. Additionally, the distribution of lesion sites, including the neck, thoracic, thoracolumbar, and lumbar segments, along with the prevalence of comorbidities, such as hypertension, diabetes, coronary heart disease, spinal cord injury, extra-pulmonary tuberculosis, and bone destruction, did not differ significantly between groups (all *P* > 0.05). Functional status indicators, including ODI and preoperative independent ambulation, were also comparable between groups (*P* > 0.05) (Table 1). Similarly, perioperative and laboratory parameters, including preoperative chemotherapy rates, intraoperative blood transfusions, and postoperative complications (e.g., pus formation, anemia, and hypoproteinemia), were comparable across groups. Perioperative factors, such as preoperative ASA scores, length of surgical incision, intraoperative blood loss, operative time, postoperative ASA scores, and time to drainage removal, showed no significant intergroup differences (*P* values ranging from 0.256 to 0.731). These findings suggest that the two groups were well-matched, supporting the reliability of the subsequent predictive analysis (Table 2).

**Table 1:** Baseline demographic, clinical, and perioperative characteristics of the modeling and validation groups

Variable	Modeling group (*n* = 170)	Validation group (*n* = 86)	Statistic	*P* value
Age (years)	48.00 (34.00, 62.00)	44.00 (29.00, 56.00)	0.187	0.312
Body mass index (kg/m^2^)	21.20 (19.80, 23.00)	21.70 (20.10, 23.50)	0.018	0.765
Gender			1.671	0.189
Male	95 (55.88%)	38 (44.19%)		
Female	75 (44.12%)	48 (55.81%)		
Smoking history			0.003	0.975
No	152 (89.41%)	77 (89.53%)		
Yes	18 (10.59%)	9 (10.47%)		
History of alcohol consumption			0.055	0.803
No	146 (85.88%)	77 (89.53%)		
Yes	24 (14.12%)	9 (10.47%)		
Lesion site			3.891	0.405
Neck segment	0 (0.00%)	2 (2.33%)		
Thoracic segment	72 (42.35%)	29 (33.72%)		
Thoracolumbar segment	19 (11.18%)	9 (10.47%)		
Waist segment	79 (46.47%)	46 (53.49%)		
Patients with prolonged hospitalization (> 21 days)	45 (26.47%)	31 (36.05%)	2.112	0.146
Hypertension			0.158	0.693
No	163 (95.88%)	80 (93.02%)		
Yes	7 (4.12%)	6 (6.98%)		
Diabetes			0.004	0.965
No	166 (97.65%)	86 (100.00%)		
Yes	4 (2.35%)	0 (0.00%)		
Coronary heart disease			0.005	0.958
No	168 (98.82%)	86 (100.00%)		
Yes	2 (1.18%)	0 (0.00%)		
Combined spinal cord injury			0.326	0.574
No	104 (61.18%)	58 (67.44%)		
Yes	66 (38.82%)	28 (32.56%)		
Combined other site tuberculosis			0.348	0.736
No	96 (56.47%)	45 (52.33%)		
Yes	74 (43.53%)	41 (47.67%)		
Combined bone destruction			0.008	0.939
No	46 (27.06%)	22 (25.58%)		
Yes	124 (72.94%)	64 (74.42%)		
Oswestry disability index	48.0 ± 11.3	46.8 ± 10.9	0.812	0.418
Preoperative independent ambulation			0.229	0.632
No	62 (36.47%)	34 (39.53%)		
Yes	108 (63.53%)	52 (60.47%)

**Table 2:** Perioperative and laboratory characteristics of the modeling and validation groups

Variable	Modeling group (*n* = 170)	Validation group (*n* = 86)	Statistic	*P* value
Preoperative chemotherapy			0.525	0.525
No	13 (7.65%)	9 (10.47%)		
Yes	157 (92.35%)	77 (89.53%)		
Intraoperative blood transfusion			0.019	0.975
No	149 (87.65%)	77 (89.53%)		
Yes	21 (12.35%)	9 (10.47%)		
Postoperative complication			0.566	0.566
No	113 (66.47%)	62 (72.09%)		
Yes	57 (33.53%)	24 (27.91%)		
Pus formation			0.793	0.793
No	21 (12.35%)	13 (15.12%)		
Yes	149 (87.65%)	73 (84.88%)		
Postoperative anemia			0.842	0.842
No	50 (29.41%)	22 (25.58%)		
Yes	120 (70.59%)	64 (74.42%)		
Postoperative hypoproteinemia			0.559	0.559
No	55 (32.35%)	22 (25.58%)		
Yes	115 (67.65%)	64 (74.42%)		
Preoperative ASA score	3.00 (2.00, 4.00)	2.00 (2.00, 3.00)	0.587	0.256
Length of surgical incision (cm)	12.00 (11.00, 15.00)	12.50 (10.50, 15.00)	0.689	0.731
Intraoperative bleeding (mL)	310.00 (205.00, 410.00)	295.00 (195.00, 395.00)	0.613	0.457
Operative time (h)	4.80 (4.05, 5.80)	4.45 (3.50, 5.85)	0.879	0.307
Postoperative ASA score	2.00 (1.00, 2.00)	2.00 (1.00, 2.00)	1.067	0.353
Time to drainage removal (d)	4.00 (3.00, 6.00)	4.00 (3.00, 6.00)	1.87	0.136

*ASA* American Society of Anesthesiologists

### Univariate analysis of potential predictors

Univariate logistic regression analysis in the modeling group identified several factors associated with prolonged hospitalization following spinal tuberculosis surgery. Among the evaluated variables, increased age emerged as a significant predictor, with each additional year associated with a 4.5% increase in the odds of prolonged hospitalization (*β* = 0.044, OR = 1.045, 95% CI 1.018–1.073, *P* = 0.002). Similarly, a higher preoperative ASA score was significantly linked to an elevated risk (*β* = 0.442, OR = 1.553, 95% CI 1.055–2.270, *P* = 0.026). Postoperative hypoproteinemia significantly impacted the outcome, tripling the odds of extended hospitalization (*β* = 1.186, OR = 3.244, 95% CI 1.300–8.100, *P* = 0.012). Additionally, patients with concomitant tuberculosis at other sites had a higher likelihood of prolonged hospitalization (*β* = 1.213, OR = 3.332, 95% CI 1.430–7.710, *P* = 0.005), as did those with combined bone destruction (*β* = 0.995, OR = 2.687, 95% CI 1.030–6.980, *P* = 0.045). The ODI, a validated measure of spinal functional impairment, showed a positive association with prolonged hospitalization; however, the result did not reach statistical significance (*β* = 0.018, OR = 1.018, 95% CI 0.987–1.051, *P* = 0.278). Similarly, patients capable of preoperative independent ambulation demonstrated a lower risk of prolonged hospitalization, but the association was not statistically significant (*β* = − 0.605, OR = 0.546, 95% CI 0.207–1.438, *P* = 0.223). Other factors, such as gender, smoking history, length of surgical incision, postoperative ASA score, time to drainage removal, and intraoperative parameters, did not reach statistical significance (Table 3).

**Table 3:** Univariate logistic regression analysis of factors associated with prolonged hospitalization in the modeling group

Factors	*β* value	Standard error value	OR value	95% CI for OR	*P* value
Length of surgical incision	− 0.003	0.057	0.997	(0.890, 1.115)	0.963
Postoperative ASA score	0.021	0.215	1.023	(0.672, 1.550)	0.935
Smoking history	0.095	0.665	1.105	(0.310, 4.100)	0.892
Time to drainage removal	0.023	0.031	1.023	(0.965, 1.085)	0.535
History of alcohol consumption	− 0.455	0.624	0.635	(0.202, 2.130)	0.478
Gender	0.415	0.423	1.515	(0.700, 3.540)	0.336
Hypertension	1.246	1.182	3.454	(0.360, 35.800)	0.292
Preoperative chemotherapy	− 1.08	0.875	0.342	(0.065, 1.860)	0.215
Intraoperative blood transfusion	0.895	0.645	2.437	(0.680, 8.700)	0.175
Postoperative complication	0.646	0.433	1.823	(0.780, 4.250)	0.173
Intraoperative bleeding	0.001	0.001	1.001	(1.000, 1.003)	0.155
Anemia	0.724	0.475	2.046	(0.830, 5.080)	0.132
Chronic disease	1.878	1.122	5.982	(0.670, 53.000)	0.117
Combined spinal cord injury	0.774	0.435	2.154	(0.930, 4.970)	0.075
Oswestry Disability Index	0.018	0.016	1.018	(0.987, 1.051)	0.278
Preoperative independent ambulation	− 0.605	0.496	0.546	(0.207, 1.438)	0.223
Pus formation	− 1.353	0.714	0.262	(0.065, 1.045)	0.058
Combined bone destruction	0.995	0.485	2.687	(1.030, 6.980)	0.045
Preoperative ASA score	0.442	0.257	1.553	(1.055, 2.270)	0.026
Postop hypoproteinemia	1.186	0.473	3.244	(1.300, 8.100)	0.012
Combined with tuberculosis of other sites	1.213	0.435	3.332	(1.430, 7.710)	0.005
Age	0.044	0.015	1.045	(1.018, 1.073)	0.002

*β* regression coefficient, *OR* odds ratio, *CI* confidence interval, *ASA* American Society of Anesthesiologists

### Independent predictors of prolonged hospitalization: multivariable stepwise regression analysis

Multivariable logistic regression analysis using a stepwise approach identified three independent predictors of prolonged hospitalization following spinal tuberculosis focus decompression, fusion, and internal fixation surgery. Specifically, increasing age was significantly associated with a higher risk of extended hospitalization, with each additional year increasing the odds by approximately 4.2% (*β* = 0.041, OR = 1.042, 95% CI 1.013–1.071, *P* = 0.005). Furthermore, the presence of concomitant tuberculosis at other sites emerged as a strong predictor, nearly tripling the risk of prolonged hospitalization (*β* = 1.062, OR = 2.875, 95% CI 1.168–7.100, *P* = 0.022). Additionally, a higher preoperative ASA score was significantly correlated with increased odds of extended hospitalization (*β* = 0.433, OR = 1.537, 95% CI 1.010–2.340, *P* = 0.046) (Table 4).

**Table 4:** Multivariable logistic regression (Stepwise) results for predicting prolonged hospitalization in the modeling group

Factors	*β* value	Standard error value	OR value	95% CI for OR	*P* value
Age	0.041	0.013	1.042	(1.013, 1.071)	0.005
Combined with tuberculosis of other sites	1.062	0.460	2.875	(1.168, 7.100)	0.022
Preoperative ASA score	0.433	0.215	1.537	(1.010, 2.340)	0.046

*β* regression coefficient, *OR* odds ratio, *CI* confidence interval, *ASA* American Society of Anesthesiologists

Prior to multivariable modeling, collinearity diagnostics did not reveal any evidence of problematic multi-collinearity among the included predictors. All VIFs were below the threshold of 5, and pairwise correlation coefficients remained within a moderate range (|*r*|< 0.7). These findings suggest that multi-collinearity did not substantially inflate the estimated odds ratios for the predictors, including age, concomitant tuberculosis at other sites, and preoperative ASA score.

### Development and validation of the prolonged hospitalization nomogram

**Fig. 1:**
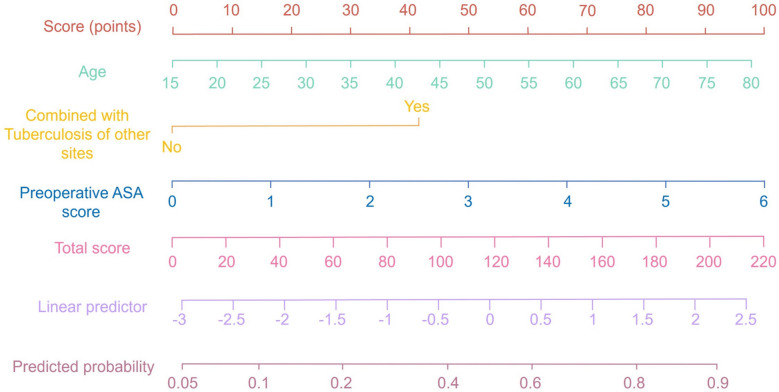
Nomogram for predicting prolonged hospitalization following spinal tuberculosis focus decompression, fusion, and internal fixation surgery

**Fig. 2:**
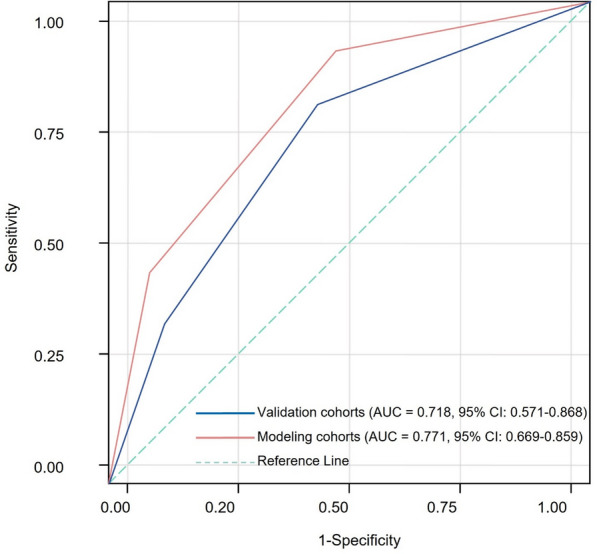
Receiver operating characteristic (ROC) curve of the nomogram, illustrating its discriminative ability for predicting prolonged hospitalization

**Fig. 3:**
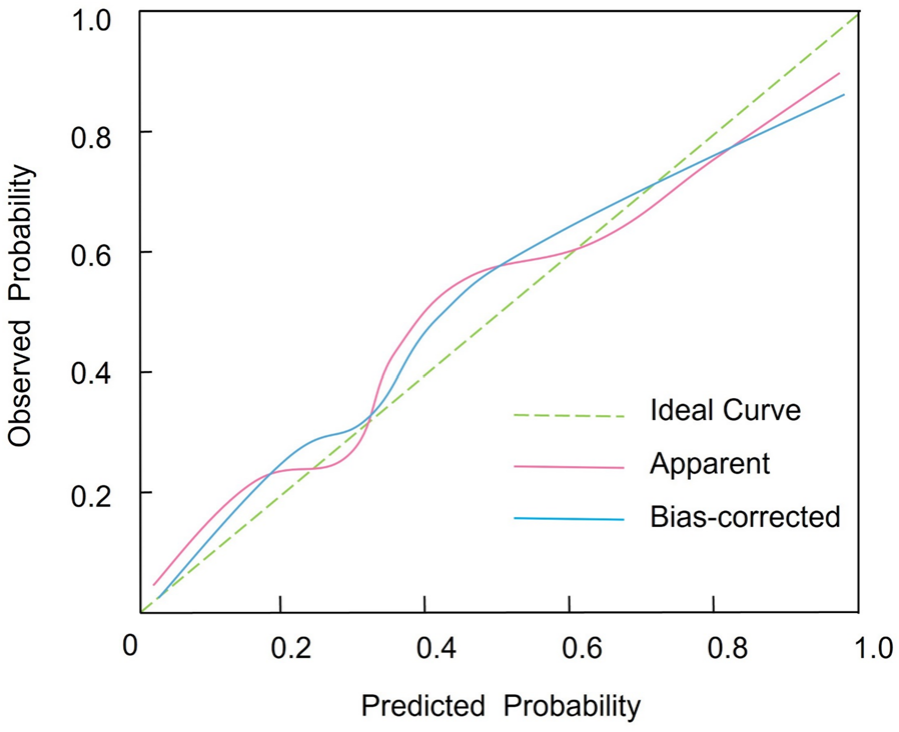
Calibration curve of the nomogram, demonstrating the agreement between predicted and observed probabilities of prolonged hospitalization

**Fig. 4:**
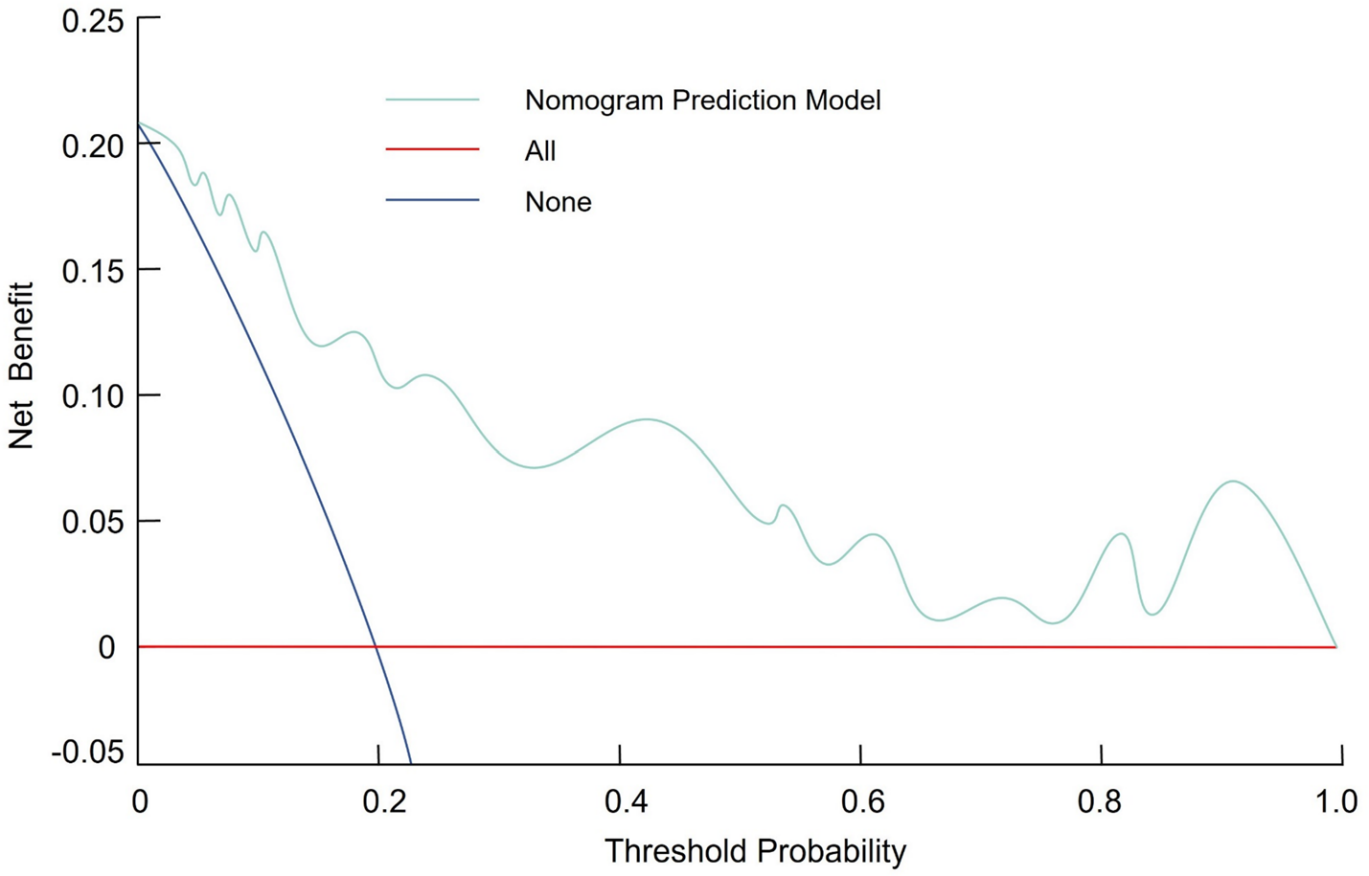
Decision curve analysis (DCA) of the nomogram, indicating its net clinical benefit across a range of threshold probabilities for prolonged hospitalization

Based on the results of the multivariable logistic regression analysis, a nomogram was constructed to predict the risk of prolonged hospitalization following spinal tuberculosis focus decompression, fusion, and internal fixation surgery. This nomogram incorporates three independent predictors—age, concomitant tuberculosis at other sites, and preoperative ASA score—with each predictor assigned an individual score according to its relative contribution. By summing these scores, clinicians can estimate the probability of prolonged hospitalization (Fig. 1). The discriminative ability of the nomogram was evaluated by constructing ROC curves. In the modeling and validation groups, the AUC was 0.771 (95% CI 0.669–0.859) and 0.718 (95% CI 0.571–0.868), respectively, indicating good predictive performance (Fig. 2). Calibration of the model was assessed using 1,000 bootstrap resamples, which yielded a corrected C index of 0.787 (95% CI 0.758–0.836). Moreover, the Hosmer–Lemeshow test demonstrated satisfactory calibration (*χ*^2^ = 2.579, *P* = 0.895) (Fig. 3). Clinical utility was further examined via DCA in both groups. The nomogram provided a net benefit superior to that of the “treat-all” or “treat-none” strategies, underscoring its potential to inform clinical decision-making regarding patient management and resource allocation (Fig. 4).

### Sensitivity analysis

Across all analyses, age, concomitant tuberculosis at other sites, and preoperative ASA score remained independently associated with prolonged hospitalization, and the direction and significance of the associations were unchanged. The corresponding odds ratios varied by less than 15% from the primary estimates, indicating stable effect sizes. Model discrimination showed minimal fluctuation, with AUC changes of < 0.03, and calibration curves remained closely aligned with the ideal reference line. Decision curve analysis (DCA) also demonstrated an almost identical net benefit profile. Collectively, these findings confirm that the predictive performance of the nomogram was robust to variations in surgical complexity.

### Sample size adequacy and model stability

In the modeling cohort, 45 patients (26.47%) experienced prolonged hospitalization (LOS > 21 days), yielding approximately 15 events per variable (EPV = 45/3 = 15) for the final multivariable logistic regression model. This EPV exceeds the commonly recommended minimum of 10 events per variable for logistic regression, suggesting that the sample size was adequate to obtain stable coefficient estimates and minimize the risk of model overfitting.

## Discussion

This study developed and evaluated a nomogram for predicting prolonged hospitalization following spinal tuberculosis surgery, specifically decompression, fusion, and internal fixation. Our analysis identified three independent predictors of extended postoperative hospital stay: patient age, concomitant tuberculosis at other sites, and a higher preoperative ASA score. The nomogram demonstrated good discrimination and calibration in both the modeling and validation groups, suggesting its potential clinical utility. By stratifying patients at high risk of prolonged hospitalization, this model enables healthcare providers to better allocate resources, optimize perioperative management, and guide decision-making. Implementing this nomogram in clinical practice could reduce unnecessary prolonged stays, improve patient outcomes, and help manage healthcare costs by offering personalized, evidence-based risk assessments for spinal tuberculosis patients undergoing surgery.

The association between increasing age and prolonged hospitalization aligns with findings in spine surgery and other surgical fields. As patients age, they often experience a decline in physiological reserve, reduced wound-healing capacity, and a higher prevalence of comorbid conditions. In our study, each additional year of age increased the odds of prolonged hospitalization by approximately 4.2% (OR = 1.042, 95% CI 1.013–1.071, *P* = 0.005). This suggests that older patients may require more intensive postoperative care and longer recovery periods. The biological rationale behind this observation may include age-related immune senescence and a decreased ability to manage the physiological stress of major surgery. Additionally, older individuals are more likely to have pre-existing conditions that complicate recovery, even if not directly captured by other variables in our study. These findings are consistent with previous studies on degenerative and infectious spinal conditions, where older age was identified as an independent risk factor for extended hospital stays [10, 19].

Another key finding of our analysis was the significant impact of concomitant tuberculosis at other sites on prolonged hospitalization. Patients with extra-spinal TB had nearly a threefold increased risk of extended hospital stay (OR = 2.875, 95% CI 1.168–7.100, *P* = 0.022). This result likely reflects the increased overall disease burden in these patients. Concomitant TB in other organs complicates the clinical picture and may require additional diagnostic procedures and multidisciplinary management, thereby delaying discharge. Furthermore, extra-spinal TB can contribute to a systemic inflammatory response and impaired nutritional status, both of which adversely affect postoperative recovery. While previous studies have primarily focused on the impact of localized spinal infection on outcomes, our finding that systemic involvement plays a crucial role in prolonging hospitalization provides new insights and highlights the importance of comprehensive preoperative assessments for patients with tuberculosis [20, 21].

The third independent predictor identified was the preoperative ASA score, which reflects overall health status and the presence of comorbid conditions. A higher ASA score was significantly associated with increased odds of prolonged hospitalization (OR = 1.537, 95% CI 1.010–2.340, *P* = 0.046). This finding supports the notion that patients with compromised general health are more susceptible to postoperative complications, delayed recovery, and extended hospital stays. The ASA score has been validated in numerous surgical studies as a predictor of morbidity and mortality, and its predictive capacity in spinal TB surgery further underscores its clinical relevance. Notably, the ASA score, as a composite measure, captures the cumulative impact of various comorbidities. While individual comorbidities, such as diabetes and hypertension, did not reach statistical significance when evaluated separately, the ASA score provided a more robust risk indicator, suggesting that the overall burden of systemic disease is a stronger predictor of prolonged hospitalization than any single medical condition [22, 23]. The nomogram model we developed integrates these three predictors into a user-friendly tool for estimating the probability of prolonged hospitalization. The model’s discriminative ability, evidenced by AUC values of 0.771 in the modeling group and 0.718 in the validation group, indicates moderate-to-good predictive performance. Internal validation using bootstrap resampling yielded a corrected C index of 0.787 (95% CI 0.758–0.836), and the Hosmer–Lemeshow test demonstrated satisfactory calibration (*χ*^2^ = 2.579, *P* = 0.895). These metrics suggest that our nomogram reliably predicts the risk of extended hospital stays based solely on preoperative data, allowing for early risk stratification and targeted intervention planning.

Chen et al.'s [24] study examines risk factors for postoperative complications in spinal tuberculosis surgery using propensity score matching, identifying albumin levels as a significant predictor. While their focus is on complications, our study specifically targets prolonged hospitalization after spinal tuberculosis surgery. Both studies highlight clinical factors influencing outcomes; however, our nomogram offers a more focused and actionable tool for predicting prolonged hospitalization, validated through ROC analysis, calibration plots, and DCA, thus providing a more precise preoperative risk stratification model. Xue et al.’s [25] research investigates the progression from pulmonary tuberculosis to spinal tuberculosis, whereas our study addresses the risk factors for prolonged hospitalization post-surgery. Although both studies use logistic regression, they differ in focus, our work is centered on surgical outcomes rather than disease progression. Our nomogram, specifically designed for preoperative risk assessment in the surgical setting, offers more immediate clinical applicability. Chen et al. [26] also developed a nomogram to predict blood transfusion risk in spinal tuberculosis surgery, evaluating the model’s performance through ROC curves, calibration, and DCA. While both studies use multivariate logistic regression to identify key predictors, our study differs in focusing on prolonged hospitalization rather than blood transfusion risk. Our nomogram provides a more comprehensive outcome and offers broader applicability, with a focus on preoperative risk factors such as the ASA score. Additionally, our study employs a multicenter validation approach, which enhances its generalizability compared to Chen et al.’s focus on blood transfusions. In summary, while the referenced studies use nomograms to predict various surgical risks, our research offers a more targeted and clinically relevant model for predicting prolonged hospitalization, supported by robust validation methods, and focuses on preoperative risk factors with broader applicability across diverse clinical settings.

The clinical implications of our findings extend beyond simple risk estimation. By offering an evidence-based tool for predicting prolonged hospitalization, the nomogram enables clinicians to identify high-risk patients before surgery and to integrate this information into individualized perioperative management. Early risk stratification can inform targeted preoperative optimization, such as nutritional support or improvement of medical comorbidities, and guide the allocation of perioperative resources, including heightened anesthetic assessment or prioritization of postoperative monitoring. High-risk patients may also benefit from early involvement of multidisciplinary teams, including infectious disease specialists, anesthesiologists, and rehabilitation services, to mitigate modifiable risks and streamline recovery pathways. Furthermore, the model supports shared decision-making by allowing clinicians to provide patients with realistic expectations regarding recovery timelines and discharge planning, which may improve satisfaction and enhance postoperative adherence. A practical example of how the nomogram can be applied in clinical settings is provided in Supplementary Material Table 1, illustrating its utility in real-world decision-making [27, 28].

This study has several limitations. First, the retrospective design introduces selection bias and limits the ability to establish causality. While the model is based on preoperative factors, it excludes intraoperative variables, such as blood loss or surgical duration, which could impact postoperative outcomes. Future research should incorporate real-time intraoperative data to improve the model’s accuracy and align it with precision medicine. Second, the single-center design restricts the generalizability of the findings. Multicenter prospective studies are needed to validate the model across diverse populations and healthcare settings. These studies should also incorporate postoperative outcomes to enhance predictive accuracy. Additionally, the study did not assess functional improvement over time, which is critical for understanding long-term recovery. Future prospective studies should include longitudinal functional outcomes, patient-reported outcomes, and quality-of-life measures to offer a more comprehensive risk assessment for spinal tuberculosis surgery patients. Future work should integrate biological markers and mechanistic insights to deepen understanding of prolonged hospitalization and refine the model’s predictive power.

## Conclusions

Advanced age, concomitant tuberculosis at other sites, and elevated preoperative ASA score were independently associated with prolonged hospitalization following spinal tuberculosis surgery. The developed nomogram, demonstrating robust discrimination and calibration, provides a practical preoperative risk assessment tool. Future prospective, multicenter studies are needed to validate and refine this model, thereby enhancing clinical decision-making.

## Supplementary Information

Supplementary Material 1.

## Data Availability

The datasets used and/or analyzed during the present study are available from the corresponding author on reasonable request.
