# Supplementary material for: Development and evaluation of a nomogram model for predicting prolonged hospitalization after spinal tuberculosis focus decompression, fusion, and internal fixation surgery
Source: Eur J Med Res. 2026 Jan 13;31:262. doi: 10.1186/s40001-025-03805-8 (PMC12888318; doi:10.1186/s40001-025-03805-8)
Supplement: Supplementary file 1 — Supplementary Material 1. [file 40001_2025_3805_MOESM1_ESM.docx]

**Supplementary Material Table 1.** Example of Nomogram Application for Predicting Prolonged Hospitalization

For illustration, consider a 52-year-old patient with concomitant tuberculosis at other sites (Yes) and a preoperative ASA score of 3.

Using the nomogram:

1. Age (52 years) → corresponds to approximately 55 points.
2. Concomitant tuberculosis at other sites (Yes) → corresponds to approximately 42 points.
3. Preoperative ASA score (3) → corresponds to approximately 50 points.

Total score → 55 + 42 + 50 = 155 points.

Predicted risk of prolonged hospitalization (>21 days) → A total score of 147 points

→Linear predictor ≈ 0.8

→Predicted probability ≈ 0.70 (70%)

This example demonstrates how clinicians can integrate preoperative characteristics into the nomogram to generate individualized risk estimates and support perioperative planning.
